# Segmental Phase Angle and Body Composition Fluctuation of Elite Ski Jumpers between Summer and Winter FIS Competitions

**DOI:** 10.3390/ijerph18094741

**Published:** 2021-04-29

**Authors:** Agnieszka Ostachowska-Gąsior, Monika Piwowar, Joanna Zając

**Affiliations:** 1 Department of Nutrition and Drug Research, Faculty of Health Sciences, Jagiellonian University Medical College, Skawinska 8 st., 31-066 Kraków, Poland; 2 Department of Bioinformatics and Telemedicine, Faculty of Medicine, Jagiellonian University Medical College, Kopernika 7e st., 31-034 Kraków, Poland; monika.piwowar@uj.edu.pl; 3 Department of Hygiene and Dietetics, Faculty of Medicine, Jagiellonian University Medical College, Kopernika 7 st., 31-034 Kraków, Poland; joanna.jankowska@uj.edu.pl

**Keywords:** body symmetry, ski jumpers, segmental phase angle, visceral fat area, BIA, winter sports, nutritional status

## Abstract

(1) Background: The purpose of this study was to observe segmental phase angle (PhA) and body composition fluctuation of elite ski jumpers. (2) Methods: In the study, 12 professional ski jumpers took part. Body composition was estimated with segmental multi-frequency bioelectrical impedance analysis. Repeated ANOVA was used to check the parameters’ variability in time. The symmetry between the right and left side of the body was verified with the *t*-test for dependent samples. Pearson’s linear correlation coefficient was calculated. (3) Results: The most stable parameter was body weight. An increase in the visceral fat area was noted, the fat-free mass dropped, and significant changes were noted in the internal and external cell water parameters. Parameters connected with water between the right and left side of the body were symmetrical. Significant correlation between PhA values and body parameters with regard to fat tissue and PhA values of the legs was noticed when PhA was measured at 50 kHz. (4) Conclusions: PhA could be considered as a ski jumper body symmetry monitoring tool. The described relationship may be useful for the assessment of body fat change, which, in the case of jumpers, is crucial. Moreover, our data suggest that segmental PhA evaluation could be a good solution for ski jumpers as a confirmation if lowered body mass and low BMI are still healthy and increase the chance for longer jumps and good performance.

## 1. Introduction

### 1.1. Importance of Body Composition for Ski Jumpers

The body composition and nutritional status of ski jumpers belong to the factors that are essential for the explosiveness required in ski jumping performance. Due to gravitational reasons, lower body mass index (BMI) definitely increases the chance for longer jump but is not a sufficient factor [1,2]. For good performance, the most important is the ability to very rapidly produce a great power output during the jump, which is achieved through a combination of the muscular strength, power, body coordination, and balance of the athlete [3,4]. Therefore, an adequate nutritional status of ski jumpers provides a chance to maintain low body weight simultaneously with a properly balanced lean-to-fat tissue ratio. For ski jumpers it is essential to monitor body composition at each stage of their preparation for competitions and during the season.

### 1.2. The Bioelectrical Impedance Analysis (BIA)

BIA remains a proper tool for this purpose. BIA is non-invasive and easy to perform and serves as a valuable tool for the evaluation of body composition and the assessment of nutritional status in either clinical patients, healthy population, or athletes [5,6,7,8]. BIA provides body composition comparable with other methods such as dual-energy X-ray absorptiometry (DXA) and hydrostatic weighing [9,10,11,12,13]. In addition, BIA is a good tool for ski jumpers, as it can be used to control the endurance of the training process in order to protect against the harmful effects of excessive endurance exercises that are part of the training to prepare jumpers for competitions [14,15]. According to recent studies, raw bioelectrical impedance parameters are useful predictors of total and extracellular pools, cellular hydration, and fluid distribution in athletes [16,17].

### 1.3. Meaning of the Phase Angle (PhA) Parameter

PhA, which can be estimated from the electrical properties of body tissue, seems to play an important role in evaluating the changes in water distribution and monitoring the hydration status of athletes regardless of sport discipline [10,18,19,20]. The PhA concept is based on the changes in resistance and reactance as an alternating current passes through the evaluated tissues. A phase shift occurring as part of the current is stored in the resistive compartments of cellular membranes [21]. Although BIA predictions of body composition often rely on population-specific equations, the PhA is estimated directly without an additional conversion to specific body parameters. Therefore, the authors highlight that the measurement of PhA depends on several biological factors, such as the quantity of cells with their respective cell membranes, cell membrane integrity, and related permeability and the amounts of extracellular and intracellular fluids [22,23,24,25]. PhA reflects the relative contributions of fluid (resistance) and cellular membranes (reactance) of the human body and is positively associated with reactance and negatively associated with resistance. Decreased cell integrity or cell death is suggested by lower PhA, while intact cell membranes are suggested by higher PhA [26,27]. That is why this parameter is considered a general marker of health, reflecting body cell mass and constituting one of the best markers of cell membrane function [28,29,30]. PhA is strongly recommended by the European Society for Clinical Nutrition and Metabolism (ESPEN) as a prognostic nutritional status measure [31]. There are considerable differences between PhA across various populations, although they share similar features in relation to age, sex, and BMI [32,33,34]. PhA values have a similar pattern that starts from infants, increases progressively up to the teenage phase, stabilizes during adult ages, and then decreases progressively in the elderly [35,36,37]. Among healthy individuals, the PhA for the whole body ranges from 5° to 7.5° [32,38]. Among trained athletes, this value rises and may reach the 8.5° [18,39] to 9.5° [40] range. A large variability in the whole-body PhA values is observed for the same sport and between various sports [41,42], but the availability of data concerning the assessment of segmental PhA is still limited [43]. Segmental (five compartments: trunk, lower and upper limbs) evaluation could be useful for monitoring the condition of the athlete preparing for a competition and for assessing differences among athletes [15,44]. As bioimpedance analysis allows us to adjust the nutrition programme within trainings and prevent unhealthy reduction of fat among ski jumpers, the aim of this study was to observe the segmental PhA and body composition fluctuations between pre-season summer training and winter competition season in the group of elite ski jumpers who represented Poland in the Ski Jumping World and Continental Cup. Moreover, our aim was to analyse the usefulness of different frequencies that are offered by segmental bioimpedance analysis. To our knowledge, this is the first study which analyses this parameter in a group of ski jumpers.

## 2. Materials and Methods

### 2.1. Study Group

Twelve members of the Polish National Team in ski jumping, world-class performers, and leaders and medallists of the Four Hills Tournament, Ski Jumping World Cup and Continental Cup, participated in the study. Their mean age was 21.7 (SD: 3.2; ranging from 17 to 29) years. All participants represented Poland in the summer and winter international competitions according to FIS competition schedule 2016/2017. The athletes were under dietician supervision, and their diet was adjusted to individual needs. The subjects were excluded from the study if they had taken medicine that might affect hydration status in the seven days prior to the test. The study was carried out in accordance with the guidelines featured in the Declaration of Helsinki. The Polish Ski Association and the athletes signed informed consent prior to the study entry, and the study protocol was approved by the Ethical Committee of the Jagiellonian University, Krakow, Poland (No. 122/6120/103/2016).

### 2.2. Measurements and Conditions

All anthropometric measurements were performed by the same operator at an ambient temperature of 22–24 °C, between 07:30 and 08:40 a.m., after fasting for at least 8 h and with an empty bladder. Body weight was measured with a medical weight scale (SECA 711), and the result was rounded to the nearest 0.1 kg. Individuals were measured barefoot, wearing minimal, light clothing. The BMI (in kg/m^2^) was calculated as body weight divided by the square meter of the height. Body composition and fluid distribution were measured with a direct segmental multi-frequency bioelectrical impedance analysis (DSM-BIA) using an impedance-meter InBody S10 (Biospace Corp., Seoul, Korea). InBody S10 is a Food and Drug Administration-approved portable version of the multi-frequency bioelectrical impedance plethysmograph body composition analyser that enables measurement in the supine position, eliminating the gravity effect of body fluid when standing. InBody S10 makes use of eight tactile electrodes: two are in contact with the palm and thumb of each hand, and two with the anterior and posterior aspects of the sole of each foot. The electrical current flows through the trunk from hand (finger) to foot (ankle) and from ankle to finger. This equipment has previously been shown to have high test–pretest reliability and accuracy and is a valid tool for the assessment of whole-body composition and for segmental lean mass measurements when validated against DXA [45]. Unlike conventional BIA equipment, which often takes only partial measurements and therefore relies upon formulas to estimate whole-body composition, the DSM-BIA technique employs the assumption that the human body is composed of five interconnected cylinders and takes direct impedance measurements from the various body compartments. An eight-point tactile electrode system is used, which separately measures the impedance of the subject’s trunk, arms, and legs at six different frequencies (1 kHz, 5 kHz, 50 kHz, 250 kHz, 500 kHz, and 1000 kHz) for each of the body segments. The spectra of electrical frequencies are used to predict the intracellular water (ICW) and extracellular water (ECW) compartments of the total body water (TBW) in the various body segments. Low-level frequencies rely on the conductive properties of extracellular fluid, whereas at high-level, the conductive properties of both ICW and ECW are instrumental. LBM is estimated as TBW (ICW+ECW)/0.73. FM is calculated as the difference between total body weight and LBM. TBW is calculated from the resistivity index Ht^2^/*R* and weight using the published bioimpedance equation of Kushner and Schoeller, modified by the manufacturer’s empirical constants [46,47]. The electrical current flows through cell membranes acting as a capacitor and resulting in a phase shift known as the geometrical phase angle, which is calculated as the arctangent of the ratio of *X*_c_ to *R* (converted to degrees) [48]. The values of PhA measured at 5 kHz, 50 kHz, and 250 kHz were taken for further considerations and calculated from the raw impedance values using the software supplied by Biospace Ltd. The measurement started with the preparation of the participants to accurately capture the impedance and PhA values. The participants were maintained in the supine position for 15 min on a non-conducting surface to avoid the effects of gravity-induced water movement. Metal and electronic devices were removed before measurement to avoid interference in the electrical current flow. The arms were slightly abducted, with elbows pronated (palms down) at about 15 degrees, and the legs were slightly abducted to the shoulder width to ensure the proper route of the current, and the skin was cleaned using wet electrolyte tissue paper to increase current conduction.

All measurements were taken at six time points:

- time point 1: (resting time) (June 2016)—2 days after completion of spring strength-endurance training series;
- time point 2—before summer FIS Grand Prix (July 2016);
- time point 3—after summer competitions (August 2016);
- time point 4, 5 (October and November 2016, respectively);
- time point 6—during the period of winter FIS Ski Jumping (World and Continental) Cup 2016/2017 (January 2017).

As a result of the measurements, the following parameters were obtained and analysed in this article: body mass (kg), body height (cm), BMI (kg/m^2^), total body water (TBW), intracellular body water (ICW), extracellular body water (ECW), ECW:TBW ratio (e_t), body cell mass (BCM), % body fat (PBF), fat (kgBF), visceral fat area (VFA), skeletal muscle mass (SMM), fat-free mass (FFM), segmental body water, segmental ECW:TBW ratio, segmental 5 kHz PA (X5 Pha), segmental 50 kHz PA (X50 Pha) and 250 kHz PA (X250 Pha). Segmental means five compartments: right arm (RA), left arm (LA), trunk (TR), right leg (RL), and left leg (LL), separately.

### 2.3. Statistical Analysis

All studied parameters were described using the mean ± standard deviation (SD) as their distributions were mostly symmetrical. Repeated ANOVA was used to check their variability in time, as standardized residuals were approximately normally distributed. Post hoc tests as well as polynomial contrasts were applied to assess the shape of changes and differences between time points. Symmetry between the right and left side of the body in segmental body water and other parameters was verified with the *t*-test for dependent samples. To investigate the effect size, Cohen’s *d* parameter was measured. A Cohen’s *d* of ±0.2 is considered a ‘small’ effect size, ±0.5 a ‘medium’ effect size, and ±0.8 a ‘large’ effect size. Pearson’s linear correlation coefficient, which determines the level of dependence between the analysed variables, was calculated. This way, variables that are significantly associated with each other were determined. For ease of interpretation, the result was presented in the form of a colour map, in which the values of the correlation coefficients are arranged on a colour scale: from dark blue, denoting the maximum positive correlation value, i.e., +1, through white, indicating no correlation 0, to dark red, indicating the maximum negative correlation value, i.e., −1. The discussion focused on Pearson’s correlation results above 0.6.

## 3. Results

The BMI did not change significantly during the study period, and the mean BMI value was 19.6 (SD: 0.56; range: 18.5–21.3 kg/m^2^). The basic anthropometric measurements and general characteristics of the participants based on BIA and segmental PhA for the whole time of observation are described in Table 1 and Appendix A.

### 3.1. Body Weight and Body Composition Fluctuation during the Period of Observation

**Figure 1:**
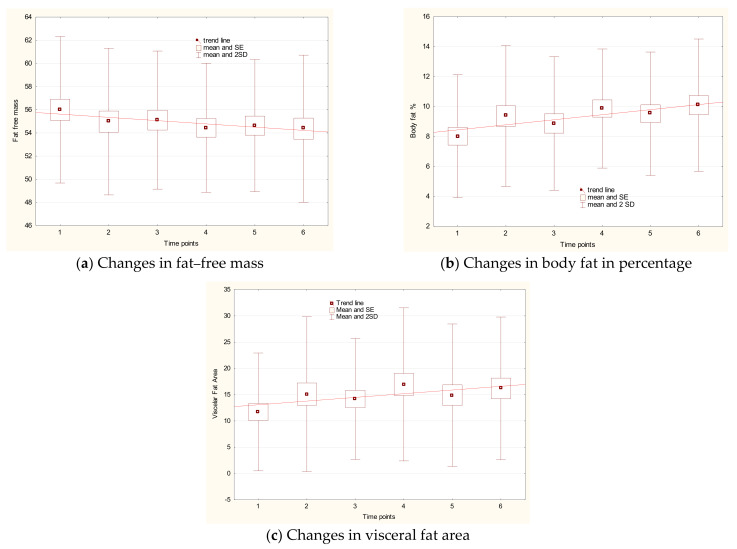
Changes in fat– free mass content (**a**), percentage of body fat (**b**), and visceral fat area (**c**) with respect to time points.

The most stable parameter during the study period was body weight. Changes in the amount of fat and in fat-free mass were noted (as % or kilograms). In general, fat tissue content rose, and the most pronounced change was observed for visceral fat area (*p*_1–6_ < 0.013). Fat-free mass, due to lowered amounts of body cell mass and skeletal muscle mass, dropped (*p*_1–6_ < 0.009). The increased body fat (% and kg) between the first and the last time point was statistically significant (*p*_1–6_ < 0.002 in both cases). The differences between the time points for chosen parameters are presented in Figure 1a–c.

**Figure 2:**
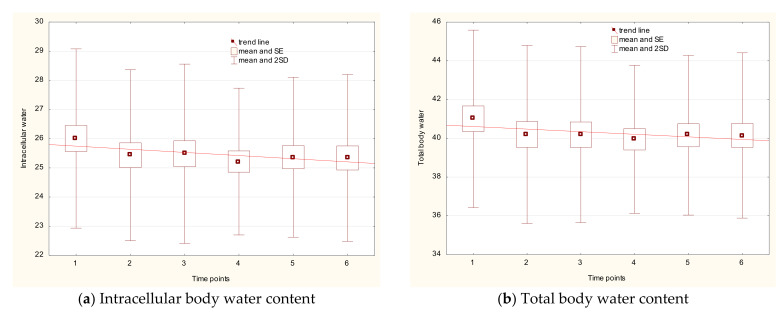

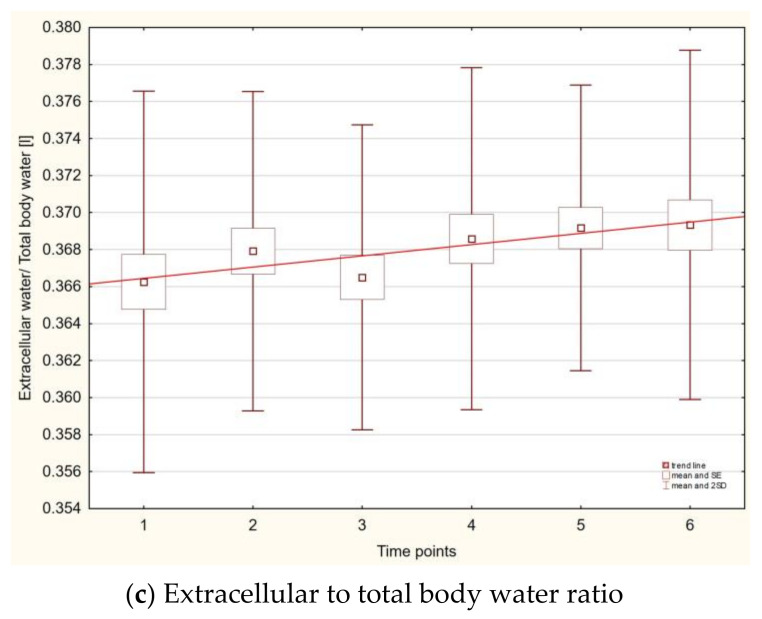
Changes in intracellular body water content (**a**), total body water content (**b**), and the ratio of extracellular body water to total body water (**c**) with respect to time points.

The total body water decreased for water parameters, ICW and TBW, whereas the parameter of ECW to TBW increased, with statistically significant differences (between the first and the last time point) of *p*_1–6_ < 0.005, *p*_1–6_ < 0.011, and *p*_1–6_ = 0.001, respectively (Figure 2a–c).

**Figure 3:**
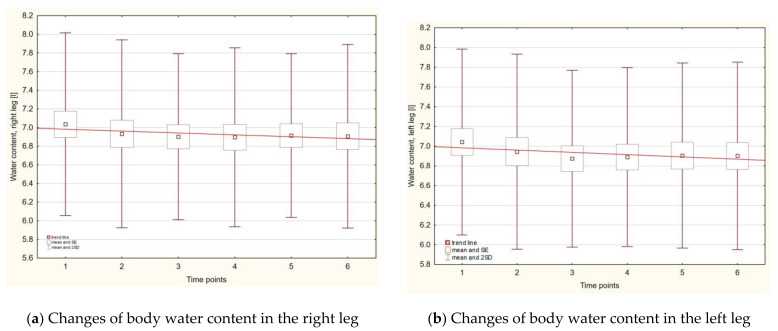
Changes of body water content in the right (**a**) and the left (**b**) leg with respect to time points.

Statistically significant changes in the parameter of water content were for both legs, between first and last measurements: *p*_1–6_ < 0.002 for right leg and *p*_1–6_ < 0.017 for the left leg (Figure 3a,b).

Water content in the legs was still lower compared with the first time point. It should be highlighted that this drop was associated with lower intracellular water content, while extracellular water content was unchanged. The highest water content was measured in the trunk, and the amount was three times greater than that in the legs. The lowest water content was noted in the arms—9 times lower than that in trunk. During the whole observation time, the amount of water between the right and the left side of the body was symmetrical for each compartment and for the ratio of extracellular body water to total body water.

### 3.2. Phase Angle Fluctuation during the Period of Observation

**Figure 4:**
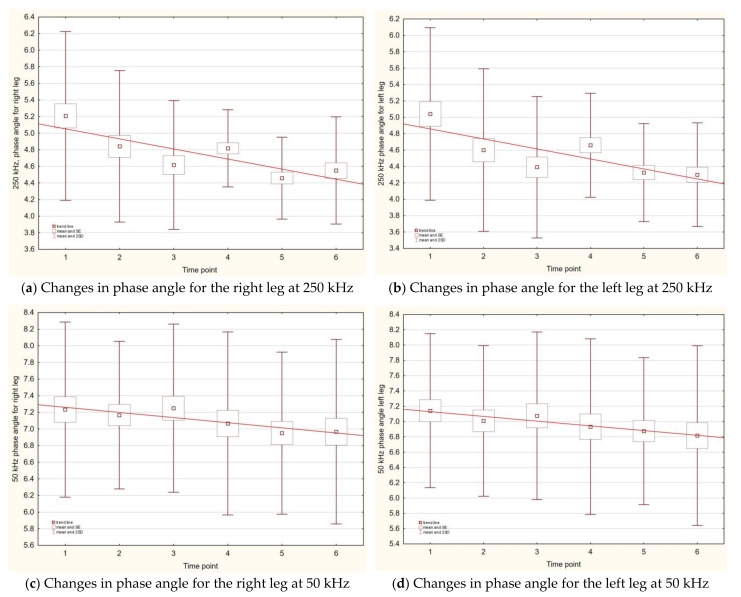
Changes in the phase angle value for both legs with respect to kHz frequency and time points.

Fluctuation in PhA values for 5, 50, and 250 kHz was observed between time points. Regardless of frequency, the highest value of PhA was for a trunk. Higher frequency was related with higher PhA values for all compartments. For both the lower limbs, the highest values of PhA were observed when using a frequency of 50 kHz, and the lowest when using 5 kHz. For all the time points, the PhA values measured with a frequency of 5 kHz for the right side of the body (arms and legs) were slightly higher than those for the left side. The highest PhA values for all body compartments (including the trunk) were noted in the third time point, that is, the end of August 2016. For a frequency of 50 kHz between each time point, substantial differences were noticed, but when comparing the starting time point and the last measurement, the differences were only observed for the right arm and the right leg. For the highest frequency (250 kHz), all body compartments had the highest PhA values in the first time point, and for subsequent time points the PhA values were dropping. For both legs, 250 kHz and 50kHz were dropping systematically, as depicted in Figure 4a–d.

### 3.3. Right-to-Left Body Side Symmetry

The results of a test analysing the significance of the differences between the right and left body parts during the study period are presented in Table 2. The upper limb PhA values for 5, 50, and 250 kHz were significantly higher for the right side of the body than those for the left, while the ECW:TBW (e_t) ratio indicated significantly higher values for the left side of the body (for the left arm as well as the left leg). The changes in the analysed parameters at different time points were inconsiderable with respect to symmetry. In the arms, the dominance of the right side of the body was visible, and PhA values were higher. The test failed to confirm the differences between the right and the left side of the body with respect to total water content and phase angle for the legs at all three frequencies.

### 3.4. Correlation Analysis for Measured Parameters

**Figure 5:**
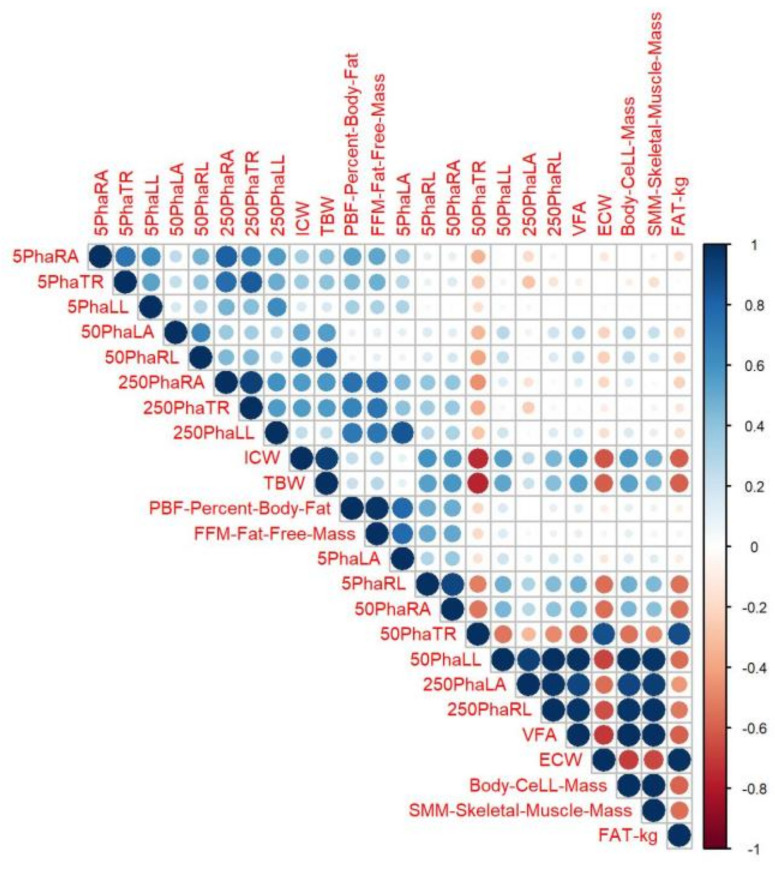
Visual representation of Pearson’s correlation coefficient for the measured parameters. More intense colours mean stronger correlation (from dark blue to light blue, positive correlation; from dark red to light red, negative correlation).

Significant correlation between PhA values and body parameters with regard to fat tissue and PhA values of the legs was noticed when PhA was measured at 50 kHz. A higher VFA parameter meant a lower PhA value for both legs (*r* > −0.75), and PhA values for the legs inversely correlated with body fat (*r* = −0.63 and *r* = −0.61; for the right and the left leg, respectively). The results of the correlation analysis are shown in Figure 5.

## 4. Discussion

Ski jumping can be classified as a speed–strength sport discipline as it requires strength, power, good balance, space orientation, and reaction abilities from professional ski jumpers [49]. For better performance, ski jumpers need to keep a body weight that can enhance their performance, and for this reason, the BMI of ski jumpers is low. As highlighted by Muller, although low weight belongs to performance determinants, it should be considered that very low weight can cause severe performance setbacks due to decreased jumping force, general weakness, reduced ability to cope with pressure, and increased susceptibility to diseases (e.g., anorexia nervosa) [50]. The BMI values of ski jumpers are changing: in 1970, the mean BMI was about 23.6 kg/m^2^, while in the first decade of the twenty-first century, a mean BMI of 19.4 kg/m^2^ was observed [2,15]. The mean BMI calculated in the present study (19.6 kg/m^2^) remains comparable and did not change significantly during the seven months of observation. This fact is inconsistent with Rønnestad’s observation, according to which the body weight of ski jumpers is reduced from pre-season to the end of the competitive season in internationally competing ski jumpers [51]. During the study period, the amount of fat tissue (visceral fat content) increased from 8.2% to 10.1%, which still adheres to the values recommended for ski jumpers and athletes of other sports in which body weight restrictions are required due to gravitational reasons, e.g., high/long jumping, long-distance running, jockeying, mountain running, and sport climbing [52]. Considering the good results of these jumpers in winter season 2016/2017, we can assume that this increased fat content should not be treated as an additional obstacle. BIA predictions of body composition depend on population-specific equations. The PhA value is not an independent variable, and circumstances such as different types of segmental analysis (e.g., foot-to-hand or direct segmental in standing position) change the results. Moreover, several biological factors, such as the quantity of cells, cell membrane integrity, and related permeability and the amounts of extracellular and intracellular fluids, affect the measured value [22,23,24]. Therefore, making comparisons of our results with other studies should be interpreted with caution. According to our results, the segmental analysis of phase angle indicated a negative statistically significant correlation between PhA for both the lower limbs (*r* > −0.75; measured at 50 kHz) and visceral fat, and only for 50 kHz frequency, PhA correlated for both legs with percentage of body fat (*r* = −0.63 and *r* = −0.61; for the right and the left leg, respectively). Our results correspond with a study by Siddiqui and co-workers [9] which indicated that PhA can be predicted from visceral fat, although the study was not based on sportsmen population and the average amount of fat in the study group was 22% (two times greater than in our population), while another study showed that the PhA of the trunk is significantly correlated with percentage of body fat [53]. During the seven months of observation, the amount of fat-free mass decreased by 1.63 kg; this decrease was caused by a decrease in skeletal muscle mass and accompanied by a decrease in the amount of body water. This change is due to the first measurement (baseline time point) being done just after the period of training, mainly composed of gym exercises, aimed at strengthening the muscles, which was followed by a resting period. Subsequent trainings preparing for the summer season are not connected exclusively with physical exertion. The change was probably partially associated with differences in body water content that fluctuate between seasons (summer and winter) [54,55]. During the study period, the water content was changing, and a decrease in water content was noticed with the most intensive drop between summer and autumn. The differences between ECW/TBW parameters were visible only when 50 kHz and 250 kHz frequencies were analysed. Though changes in the proportion of water content may be evidence of malnutrition, according to the literature, these changes are probably associated with the increase in visceral fat content and change in the type of exercise [21,56]. It is worth highlighting that the most pronounced changes are associated with the lower limbs, probably because the legs are the most metabolically active body parts of ski jumpers [3,57]. As the literature data associated with water monitoring in each body segment, as well as detailed studies monitoring body parameters in regular time spans during the training of ski jumpers, are scanty, our results cannot be compared with other studies, to draw firm conclusions. The BIA method and PhA value are an accepted marker of cells’ healthiness and an important tool in assessing nutritional status in any situation, being superior to anthropometric and biochemical methods [58]. On the basis of the literature, it is known that when a drop in BMI is not accompanied by a decrease in PhA, we can assume that there is no threat of undernutrition. In constitutionally lean people and ballet dancers, despite lower BMI, phase angle is not decreased. The explanation of this fact is body composition—less fat and more muscle mass as a result of workout [43,59]. For ski jumpers who practice lower BMI simultaneously with a lower percentage of body fat, the same tendency may be expected. Higher BMI is associated with increasing PhA in more cells, but only up to a BMI of 30 kg/m^2^. Interestingly, at BMI > 40 kg/m^2^, an inverse relationship with PhA is observed. This has been attributed to increased tissue hydration or a pathological fluid overload [23]. Our data suggest that in the case of phase angle monitoring among ski jumpers, 50 kHz can be more useful than other frequencies, as this PhA value is correlated, for both legs, with VFA and percentage of body fat, with the following tendency: the lower the PhA value, the higher the fat proportion. The described relationship may be useful for a rough assessment of body fat change, which, in the case of jumpers, is crucial. Moreover, our data suggest that phase angle could be potentially useful in monitoring the symmetry of ski jumpers’ legs.

### 4.1. Practical Application

From a practical point of view, at the present time the measurement of PhA, especially PhA monitoring at 50 kHz, is a promising approach to evaluate muscle quality in athletes and the nutritional status and healthiness of body cells, which should be controlled at all stages of the ski jumper’s preparation by BIA. We have to agree that there are more precise methods for the evaluation of body composition, such as DXA, but still they are invasive and cannot be repeated frequently, while BIA is non-invasive and fast and provides reliable results, as long as the measurement conditions are always the same and there are no ambient factors that could affect the amount of body water.

### 4.2. Strengths and Limitations

The main strength of the present work is that it presents a detailed analysis and description of a specific, selected population of high-performing ski jumpers at different time points throughout their training season. The main limitation of our study remains the very small number of participants, though we aimed to focus on a specific group of Olympic ski jumpers. Moreover, there are limited studies reporting changes in DSM-BIA parameters during a training season, which makes comparisons more difficult and lowers the strength of our conclusions.

## 5. Conclusions

For ski jumpers, the differences in body composition of each compartment are the result of special training, and the segmental MFBIA seems to be a good tool to monitor these changes repeatedly during the season to ensure that lowered body mass is the consequence of a fat mass decrease and low BMI is still healthy and increases the chance for longer jumps and good performance. Therefore, segmental PhA values can help monitor progress and healthiness during the training season in terms of body fat content, visceral fat area, water content, and body symmetry. In addition, we suggest that for ski jumpers, PhA monitoring at 50 kHz can be more useful than at other frequencies. Although further studies are needed to scrutinize how changes, observed during the study, fluctuate over time, the results of the measurements are still informative, at least regarding the information about intact cell membranes. Understanding BIA as a tool that monitors body components, including segmental phase angle, during ski jumpers’ training remains an open area for research.

## Figures and Tables

**Table 1:** General characteristics of the participants based on BIA and segmental PhA measurements.

Parameter	Mean	SD	Min	Max
Height [m]	1.76	0.033	1.70	1.80
Weight [kg]	60.47	2.489	56.40	66.47
BMI [kg/m^2^]	19.58	0.570	18.54	21.28
ICW (intracellular body water)	25.48	1.409	23.50	29.00
ECW (extracellular body water)	14.80	0.739	13.60	16.60
TBW (total body water)	40.28	2.115	37.10	45.6
h2oRA (water content, right arm)	2.29	0.177	1.97	2.70
h2oLA (water content, left arm)	2.27	0.168	1.92	2.70
h2oTR (water content, trunk)	18.77	0.975	17.00	20.90
h2oRL (water content, right leg)	6.93	0.461	6.36	8.10
h2oLL (water content, left leg)	6.93	0.456	6.32	8.10
e_t_Total (ECW:TBW ratio; total)	0.37	0.004	0.36	0.40
e_tRA (ECW:TBW ratio; right arm)	0.37	0.004	0.36	0.40
e_tLA (ECW:TBW ratio; left arm)	0.37	0.004	0.36	0.40
e_tTR (ECW:TBW ratio; trunk)	0.37	0.005	0.36	0.40
e_tRL (ECW:TBW ratio; right leg)	0.37	0.005	0.36	0.40
e_tLL (ECW:TBW ratio; left leg)	0.37	0.005	0.36	0.40
BCM (Body Cell Mass) in kg	36.45	2.040	33.20	41.50
SMM (Skeletal Muscle Mass) in kg	31.18	1.855	28.20	35.80
FFM (Fat-Free Mass) in kg	54.92	2.968	49.90	62.30
Fat in kg	5.61	1.287	2.20	8.00
PBF (Percentage of body fat)	9.29	2.187	3.40	13.60
VFA (Visceral Fat Area) in cm^2^	14.84	6.611	5.00	30.00
X5PhaRA (5 kHz PA; right arm)	2.58	0.220	2.00	3.00
X5PhaLA (5 kHz PA; left arm)	2.47	0.240	1.90	3.00
X5PhaTR (5 kHz PA; trunk)	3.85	0.294	3.10	4.70
X5PhaRL (5 kHz PA; right leg)	3.07	0.443	2.30	6.00
X5PhaLL (5 kHz PA; left leg)	2.97	0.279	2.30	3.50
X50PhaRA (50 kHz PA; right arm)	6.23	0.438	5.20	7.10
X50PhaLA (50 kHz PA; left arm)	6.00	0.512	4.70	7.00
X50PhaTR (50 kHz PA; trunk)	9.45	0.974	7.60	12.70
X50PhaRL (50 kHz PA; right leg)	7.11	0.509	6.10	8.20
X50PhaLL (50 kHz PA; left leg)	6.98	0.526	5.90	8.10
X250PhaRA (250 kHz PA; right arm)	6.11	0.624	5.00	7.80
X250PhaLA (250 kHz PA; left arm)	5.93	0.677	4.80	7.60
X250PhaTR (250 kHz PA; trunk)	9.34	3.511	4.00	20.70
X250PhaRL (250 kHz PA; right leg)	4.75	0.438	3.90	6.20
X250PhaLL (250 kHz PA; left leg)	4.55	0.471	3.80	6.00

**Table 2:** Differences between left and right body parts with respect to total body water, the ratio of extracellular body water to total body water, and the phase angle at 5 kHz, 50 kHz, and 250 kHz.

	Mean	SD	95% Confidence Interval	Statistical Significance *p*	Effect Size	
					Cohen’s *d*	95%CI
h2oRA–h2oLA	0.006	0.0491	−0.025–0.037	0.688	−	
h2oRL–h2oLL	−0.006	0.0675	−0.049–0.037	0.770	−	
e_tRA–e_tLA *	−0.001	0.0017	−0.002–−0.0001	0.041	−0.65	[−0.91, −0.40]
e_tRL–e_tLL *	−0.003	0.0024	−0.004–−0.001	0.003	−1.22	[−1.54, −0.92]
5kHzPhaRA–5kHzPhaLA *	0.150	0.1679	0.043–0.257	0.010	0.65	[0.40, 0.91]
5kHzPhaRL–5kHzPhaLL	0.100	0.2132	−0.035–0.235	0.132	−	
50kHzPhaRA–50kHzPhaLA *	0.292	0.1975	0.166–0.417	0.000	1.22	[0.92, 1.54]
50kHzPhaRL–50kHzPhaLL	0.092	0.1832	−0.025–0.208	0.111	−	
250kHzPhaRA–250kHzPhaLA *	0.150	0.1382	0.062–0.238	0.003	1.07	[0.78, 1.37]
250kHzPhaRL–250kHzPhaLL	0.167	0.3114	−0.031–0.365	0.091	−	

* No significant difference in body symmetry between right and left body parts.

## Data Availability

Restrictions apply to the availability of this data. Data become the property of the Polish Ski Association and are available from the corresponding author with the permission of Polish Ski Association.

## Appendix A

**Table A1:** Fluctuation in the analysed parameters (table).

Changes in Analysed Parameters during the Whole Time of Observation								
	Time Point						*p*	*p* for Pairwise Comparisons
	1	2	3	4	5	6		
	Value ± SD	Value ± SD	Value ± SD	Value ± SD	Value ± SD	Value ± SD		
**BMI**	19.71 ± 0.63	19.63 ± 0.60	19.51 ± 0.63	19.60 ± 0.41	19.45 ± 0.45	19.57 ± 0.73	0.295	
**ICW**	26.01 ± 1.54	25.43 ± 1.47	25.48 ± 1.54	25.22 ± 1.26	25.37 ± 1.37	25.34 ± 1.43	<0.001	*p*_12_ = 0.009; *p*_13_ = 0.013; *p*_14_ = 0.001; *p*_15_ = 0.007; *p*_16_ = 0.005
**ECW**	15.00 ± 0.79	14.77 ± 0.86	14.71 ± 0.75	14.73 ± 0.68	14.79 ± 0.72	14.81 ± 0.75	0.022	*p*_12_ = 0.027; *p*_13_ = 0.002; *p*_14_ = 0.010; *p* for quadratic trend: <0.001
**TBW**	41.01 ± 2.29	40.20 ± 2.30	40.19 ± 2.27	39.95 ± 1.91	40.16 ± 2.06	40.15 ± 2.13	0.005	*p*_12_ = 0.011; *p*_13_ = 0.005 ; *p*_14_ = 0.002; *p*_15_ = 0.013; *p*_16_ = 0.011; *p* for linear trend: 0.022
**h2oRA**	2.31 ± 0.18	2.29 ± 0.2	2.28 ± 0.2	2.26 ± 0.16	2.31 ± 0.18	2.32 ± 0.18	0.021	*p*_14_ = 0.026; *p*_36_ = 0.020; *p*_45_ = 0.005; *p*_46_ = 0.002; *p* for quadratic trend: 0.001
**h2oLA**	2.3 ± 0.19	2.27 ± 0.2	2.24 ± 0.18	2.25 ± 0.15	2.28 ± 0.15	2.28 ± 0.16	0 .103	
**h2oTR**	18.89 ± 1.05	18.79 ± 1.14	18.64 ± 1.08	18.64 ± 0.87	18.82 ± 0.92	18.86 ± 0.94	0.083	
**h2oRL**	7.04 ± 0.49	6.93 ± 0.5	6.9 ± 0.45	6.90 ± 0.48	6.92 ± 0.44	6.91 ± 0.49	0.016	*p*_12_ = 0.040; *p*_13_ = 0.022; *p*_14_ = 0.002; *p*_15_ = 0.030; *p*_16_ = 0.002 *p* for linear trend: 0.007
**h2oLL**	7.04 ± 0.47	6.94 ± 0.49	6.87 ± 0.45	6.89 ± 0.45	6.90 ± 0.47	6.90 ± 0.48	0.003	*p*_12_ = 0.038; *p*_13_ = 0.005; *p*_14_ = 0.001; *p*_15_ = 0.030; *p*_16_ = 0.017 *p* for linear trend: 0.032
**e_t**	0.366 ± 0.005	0.368 ± 0.004	0.367 ± 0.004	0.369 ± 0.005	0.369 ± 0.004	0.369 ± 0.005	<0.001	*p*_12_ = 0.017; *p*_14_ = 0.006; *p*_15_ < 0.001; *p*_16_ = 0.001; *p*_25_ = 0.006; *p*_35_ < 0.001; *p*_36_ = 0.032 *p* for linear trend: <0.001
**e_tRA**	0.367 ± 0.005	0.369 ± 0.004	0.369 ± 0.003	0.370 ± 0.004	0.371 ± 0.003	0.371 ± 0.004	<0.001	*p*_12_ = 0.019; *p*_14_ = 0.005; *p*_15_ = 0.001; *p*_16_ = 0.005; *p*_35_ = 0.002; *p*_36_ = 0.002; *p* for linear trend: <0.001
**e_tLA**	0.369 ± 0.005	0.371 ± 0.005	0.370 ± 0.004	0.371 ± 0.004	0.372 ± 0.003	0.372 ± 0.005	0.001	*p*_12_ = 0.011; *p*_14_ = 0.019; *p*_15_ = 0.008; *p*_16_ = 0.017; *p*_23_ = 0.025; *p*_34_ = 0.010; *p*_35_ = 0.003; *p*_36_ = 0.036; *p* for linear trend: 0.014
**e_tTR**	0.365 ± 0.006	0.368 ± 0.004	0.366 ± 0.004	0.368 ± 0.005	0.369 ± 0.004	0.369 ± 0.005	<0.001	*p*_12_ = 0.006; *p*_14_ = 0.003; *p*_15_ = 0.004; *p*_16_ = 0.001; *p*_23_ = 0.022; *p*_34_ = 0.004; *p*_35_ = 0.001; *p*_36_ = 0.002; *p* for linear trend: <0.001
**e_tRL**	0.366 ± 0.006	0.366 ± 0.005	0.366 ± 0.005	0.367 ± 0.006	0.368 ± 0.005	0.368 ± 0.006	0.038	*p*_15_ = 0.046; *p*_25_ = 0.046; *p*_35_ = 0.015; *p*_36_ = 0.026; *p* for linear trend: 0.026
**e_tLL**	0.369 ± 0.005	0.369 ± 0.005	0.368 ± 0.005	0.370 ± 0.006	0.370 ± 0.005	0.370 ± 0.006	0 .156	
**BCM**	37.23 ± 2.18	36.48 ± 2.12	36.59 ± 2.06	36.11 ± 1.93	36.23 ± 1.97	36.03 ± 2.16	<0.001	*p*_12_ = 0.019; *p*_13_ = 0.019; *p*_14_ < 0.001; *p*_15_ = 0.002; *p*_16_ = 0.007; *p*_34_ = 0.031; *p* for linear trend: 0.005
**SMM**	31.89 ± 1.98	31.22 ± 1.94	31.32 ± 1.89	30.88 ± 1.74	30.98 ± 1.77	30.81 ± 1.97	<0.001	*p*_12_ = 0.019; *p*_13_ = 0.018; *p*_14_ = 0.001; *p*_15_ = 0.002; *p*_16_ = 0.007; *p*_34_ = 0.029; *p* for linear trend: 0.004
**FFM**	56.00 ± 3.16	54.98 ± 3.17	55.11 ± 2.98	54.44 ± 2.79	54.63 ± 2.85	54.37 ± 3.17	<0.001	*p*_12_ = 0.025; *p*_13_ = 0.016; *p*_14_ = 0.001; *p*_15_ = 0.002; *p*_16_ = 0.009; *p* for linear trend: 0.005
**FAT**	4.87 ± 1.15	5.67 ± 1.41	5.36 ± 1.34	5.93 ± 1.15	5.76 ± 1.22	6.09 ± 1.30	<0.001	*p*_12_ = 0.009; *p*_14_ = 0.001; *p*_15_ = 0.005; *p*_16_ = 0.002; *p*_34_ = 0.025; *p*_36_ = 0.011 *p* for linear trend: 0.003
**PBF**	8.02 ± 2.05	9.37 ± 2.35	8.87 ± 2.23	9.87 ± 1.99	9.52 ± 2.06	10.09 ± 2.21	<0.001	*p*_12_ = 0.009 ; *p*_14_ = 0.001; *p*_15_ = 0.004; *p*_16_ = 0.002; *p*_34_ = 0.012; *p*_36_ = 0.007; *p* for linear trend: 0.002
**VFA**	11.71 ± 5.61	15.10 ± 7.36	14.18 ± 5.76	16.96 ± 7.29	14.9 ± 6.77	16.19 ± 6.79	0.002	*p*_12_ = 0.032; *p*_14_ = 0.002; *p*_16_ = 0.013; *p*_34_ = 0.038; *p*_45_ = 0.009; *p* for linear trend: 0.024
**5PhaRA**	2.64 ± 0.24	2.62 ± 0.24	2.66 ± 0.19	2.53 ± 0.27	2.50 ± 0.16	2.55 ± 0.20	0.059	
**5PhaLA**	2.49 ± 0.27	2.47 ± 0.27	2.53 ± 0.26	2.43 ± 0.28	2.44 ± 0.17	2.46 ± 0.21	0.547	
**5PhaTR**	3.77 ± 0.34	3.88 ± 0.35	3.98 ± 0.23	3.83 ± 0.26	3.85 ± 0.25	3.79 ± 0.33	0.177	
**5PhaRL**	2.99 ± 0.24	2.99 ± 0.28	3.21 ± 0.23	3.02 ± 0.32	3.01 ± 0.22	3.23 ± 0.92	0.355	
**5PhaLL**	2.89 ± 0.24	2.97 ± 0.28	3.16 ± 0.22	2.94 ± 0.32	2.93 ± 0.23	2.93 ± 0.34	0.004	*p*_13_ < 0.001; *p*_23_ = 0.024; *p*_34_ = 0.002; *p*_35_ = 0.005; *p*_36_ = 0.017; *p* for quadratic trend: 0.011
**50PhaRA**	6.40 ± 0.50	6.23 ± 0.45	6.29 ± 0.40	6.23 ± 0.44	6.08 ± 0.38	6.15 ± 0.48	<0.001	*p*_12_ = 0.012; *p*_14_ = 0.031; *p*_15_ = 0.001; *p*_16_ = 0.004; *p*_25_ = 0.010; *p*_35_ < 0.001; *p*_36_ = 0.037; *p*_45_ = 0.034; *p* for linear trend: 0.001
**50PhaLA**	6.11 ± 0.61	5.97 ± 0.56	6.09 ± 0.49	6.03 ± 0.54	5.87 ± 0.44	5.93 ± 0.49	0.014	*p*_15_ = 0.013; *p*_35_ = 0.002; *p*_36_ = 0.014; *p*_45_ = 0.037;
**50PhaTR**	9.82 ± 1.02	8.98 ± 0.56	10.03 ± 1.06	9.27 ± 0.91	9.28 ± 0.70	9.34 ± 1.22	0.005	*p*_23_ < 0.001; *p*_35_ < 0.003; *p*_34_ = 0.005; *p*_36_ = 0.012; *p*_12_ = 0.012
**50PhaRL**	7.23 ± 0.53	7.17 ± 0.44	7.25 ± 0.51	7.07 ± 0.55	6.95 ± 0.49	6.97 ± 0.55	<0.001	*p*_15_ = 0.008; *p*_16_ = 0.001; *p*_25_ = 0.017; *p*_26_ = 0.018; *p*_35_ = 0.001; *p*_36_ = 0.008; *p* for linear trend: 0.001
**50PhaLL**	7.14 ± 0.50	7.01 ± 0.49	7.08 ± 0.55	6.93 ± 0.57	6.88 ± 0.48	6.82 ± 0.59	<0.001	*p*_12_<0.039; *p*_14_ = 0.001; *p*_15_ = 0.001; *p*_16_ = 0.001; *p*_25_ = 0.039; *p*_26_ = 0.019; *p*_35_ = 0.013; *p*_36_ = 0.021 *p* for linear trend: <0.001
**250PhaRA**	6.70 ± 0.76	6.00 ± 0.48	6.14 ± 0.45	6.07 ± 0.46	5.75 ± 0.45	5.98 ± 0.73	<0.001	*p*_12_ < 0.005; *p*_13_ < 0.026; *p*_14_ = 0.001; *p*_15_<0.001; *p*_16_ = 0.011; *p*_35_ = 0.018; *p*_45_ = 0.012 *p* for linear trend: 0.001
**250PhaLA**	6.55 ± 0.79	5.83 ± 0.55	6.03 ± 0.51	5.88 ± 0.57	5.53 ± 0.49	5.79 ± 0.75	<0.001	*p*_12_ < 0.008; *p*_14_ = 0.002; *p*_15_ < 0.001; *p*_16_ = 0.011; *p*_25_ = 0.037; *p*_25_ = 0.012; *p*_45_ = 0.008 *p* for linear trend: 0.001
**250PhaTR**	12.43 ± 4.22	6.89 ± 1.68	10.48 ± 2.64	8.23 ± 3.10	8.48 ± 2.80	9.53 ± 3.74	<0.001	*p*_12_ < 0.001; *p*_14_ < 0.001; *p*_15_ = 0.006; *p*_23_ = 0.007; *p*_25_ = 0.022; *p* for quadratic trend: 0.017
**250PhaRL**	5.21 ± 0.51	4.84 ± 0.46	4.62 ± 0.39	4.82 ± 0.23	4.46 ± 0.25	4.55 ± 0.32	<0.001	*p*_13_ = 0.001; *p*_14_ = 0.004; *p*_15_ < 0.001; *p*_16_ < 0.001; *p*_25_ = 0.011; *p*_26_ = 0.020; *p*_34_ = 0.028; *p*_45_ = 0.001; *p*_46_ = 0.015 *p* for linear trend: <0.001
**250PhaLL**	5.04 ± 0.53	4.60 ± 0.50	4.39 ± 0.43	4.66 ± 0.32	4.33 ± 0.30	4.30 ± 0.32	<0.001	*p*_13_ = 0.002; *p*_14_ = 0.003; *p*_15_ < 0.001; *p*_16_ < 0.001; *p*_25_ = 0.033; *p*_34_ = 0.023; *p*_45_ = 0.007; *p*_46_ = 0.003 *p* for linear trend: <0.001

Index of abbreviations: Total body water (TBW), intracellular body water (ICW), extracellular body water (ECW), ECW:TBW ratio (e_t), body cell mass (BCM), % body fat (PBF), fat (kgBF), visceral fat area (VFA), skeletal muscle mass (SMM), fat-free mass (FFM), segmental body water (h2o), segmental ECW:TBW ratio (e_t), segmental 5 kHz phase angle (PA) (5 Pha), segmental 50 kHz PA (50 Pha), and 250 kHz PA (250 Pha). Segmental means 5 compartments: right arm (RA), left arm (LA), trunk (TR), right leg (RL), and left leg (LL), respectively.
